# Autonomous Motivation Trajectory Following Adoption of a Team-Based Gamification App Among Adults With Diabetes: 1-Year Formative Longitudinal Study

**DOI:** 10.2196/87236

**Published:** 2026-02-19

**Authors:** Satoshi Inagaki, Kenji Kato, Tomokazu Matsuda, Kozue Abe, Hisafumi Yasuda

**Affiliations:** 1 School of Data Science Nagoya City University Nagoya, Aichi Japan; 2 Graduate School of Health Sciences Kobe University Kobe, Hyōgo Japan; 3 Faculty of Nursing Kobe Women's University Kobe, Hyogo Japan; 4 Matsuda Diabetes Clinic Kobe, Hyogo Japan

**Keywords:** smartphone, health apps, eHealth, diabetes mellitus, digital health, social support, self-care, gamification, patient participation, consumer health information

## Abstract

**Background:**

Autonomous motivation, grounded in self-determination theory, is important for sustaining diabetes self-care behaviors. Although mobile health interventions, gamification, and peer support are increasingly used to enhance motivation in diabetes care, evidence on how motivation evolves over time remains limited. Specifically, it is unclear whether motivational change follows a linear pattern or a nonlinear trajectory, such as an initial increase followed by a subsequent decline. Clarifying these temporal patterns is critical for informing the design of adaptive diabetes self-care interventions.

**Objective:**

The objective of this study was to characterize the 1-year developmental trajectory of autonomous motivation following the real-world introduction of a commercially available team-based gamification app.

**Methods:**

This prospective, single-arm longitudinal study involved adults with diabetes (predominantly type 2) recruited from outpatient clinics in Japan. Participants were instructed to use a team-based gamification app designed to promote desirable habits through peer support and social comparison for at least 7 days. The primary outcome, autonomous motivation, was assessed using the Treatment Self-Regulation Questionnaire–Autonomous Motivation subscale (TSRQ-AM; score range 7-49) at baseline, 6 weeks, 6 months, and 1 year. Secondary measures included hemoglobin A_1c_ (HbA_1c_), body weight, triglycerides, and psychological scales (eg, Self-Efficacy Scale for Diabetes Self-Care, Summary of Diabetes Self-Care Activities, Problem Areas in Diabetes scale, and World Health Organization–Five Well-Being Index). To analyze the trajectory, we used linear mixed-effects models with random intercepts for participants. The final model included fixed effects for time (as both linear and quadratic terms), age, sex, employment status, family structure, baseline BMI, and baseline HbA_1c_.

**Results:**

Of 32 consenting participants, 29 (90.6%) were included in the primary analysis; clinical data at 1 year were available for 26 (81.3%) participants. In exploratory analyses, mean TSRQ-AM scores increased from baseline (37.4, SD 7.9) to 6 months (39.5, SD 7.4; Cohen *d*=0.47). Over the 1-year period, body weight decreased significantly (*b*=−0.39; *P*=.01), whereas HbA_1c_ (*P*=.40) and triglycerides (*P*=.14) showed no significant changes. The TSRQ-AM score showed a significant nonlinear change over time. A model including a quadratic time term fit significantly better than a linear-only model (*χ*^2^_1_=4.1; *P*=.04), with a significant quadratic effect (*b*=−7.26; *P*=.045), indicating an inverted U-shaped trajectory peaking at 6 months. Higher baseline BMI was associated with lower TSRQ-AM scores (*b*=−1.00; *P*=.001).

**Conclusions:**

This formative study provides preliminary evidence of a nonlinear, 1-year trajectory of autonomous motivation following the introduction of a team-based app. The observed curvilinear pattern suggests that autonomous motivation during the intervention may peak at around 6 months, underscoring the importance of adaptive intervention designs to maintain engagement over time. The accompanying reduction in body weight suggests potential physiological relevance that warrants further investigation in controlled studies.

**Trial Registration:**

UMIN Clinical Trials Registry UMIN000044874; https://tinyurl.com/59bzb68k

## Introduction

Autonomous motivation is a critical determinant of sustained diabetes self-care, and its importance has been emphasized in numerous studies [1-3]. According to self-determination theory (SDT) [4], autonomous motivation—engaging in behaviors out of personal interest or values—is linked to better long-term adherence, whereas controlled motivation (pressure or obligation) is less sustainable. Therefore, fostering autonomous motivation is a key goal in diabetes self-care interventions [3,4].

Both peer support and mobile health (mHealth) interventions have been proposed as ways to enhance patient motivation [5,6]. For instance, some peer support programs have been shown to have a positive impact on self-care and psychological well-being [7,8], but they often encounter logistical challenges that restrict widespread access [9]. In contrast, mHealth interventions can provide support regardless of time and place. Studies have found mHealth to have an independent effect on hemoglobin A_1c_ (HbA_1c_) and self-care behaviors [10,11]. The use of mHealth in diabetes care continues to expand. In particular, gamification approaches are expected to sustain motivation by leveraging social comparison and reward mechanisms [12]. However, the long-term motivational effects of prescribing such apps as part of routine diabetes care remain unclear. Furthermore, the temporal trajectory of these motivational changes beyond a few weeks is largely unknown. It is unclear whether psychological outcomes improve linearly or follow a more complex, nonlinear path (eg, peaking and then declining). Understanding this pattern is critical for designing interventions that can sustain engagement.

In a prior report, we introduced a commercially available team-based habit-forming app that incorporates gamification as a means to integrate peer support and mHealth [13]. A preliminary qualitative analysis conducted at 6 weeks revealed that approximately 80% of participants found the app helpful for self-care, while some also reported mixed experiences. Importantly, some participants expressed negative impressions stemming from conflicts within their teams. These findings indicate preliminary signs of early benefits, but it is not yet clear whether such motivational gains can be maintained over the long term.

Building on these findings, the purpose of this study was to investigate the 1-year developmental trajectory of autonomous motivation following the implementation of a team-based gamification app, with the hypothesis that autonomous motivation would increase over time. To reflect real-world conditions, participants were only instructed to try the app for at least 7 days and were free to discontinue thereafter. The primary outcome of interest was autonomous motivation, a key psychological factor for sustaining diabetes self-care.

## Methods

### Research Design and Participants

This prospective, single-arm, hypothesis-generating longitudinal study was conducted between 2022 and 2023. Participants were adults with type 1 or type 2 diabetes who voluntarily responded to recruitment posters displayed at outpatient clinics.

### Intervention

Participants were instructed to download and use Minchalle (A10 Lab Inc), a commercially available team-based gamification app designed to promote the formation of desirable habits. Grounded in social cognitive theory, the app facilitates behavior change through small anonymous groups (up to 5 members) that foster social support, social comparison, and accountability. The app has been widely adopted, with more than 1.6 million downloads. Users share daily progress via photos on team chats, provide mutual feedback, and receive in-app rewards such as coins and badges for task completion, reinforcing sustained engagement. Inactive members are automatically removed, emphasizing active participation and shared responsibility.

Participants were required to join a team and try using the app for at least 7 days; thereafter, continued use was optional. Teams were selected according to personal preference (eg, exercise-oriented or daily weight–tracking teams), and no diabetes-specific team assignment was mandated. To ensure that all participants could meet the initial 7-day requirement under equal conditions, they were provided with researcher-funded access to the app’s premium features at no cost. Detailed app features have been described in our previous study [13].

### Measures

The primary outcome was autonomous motivation, assessed using the Treatment Self-Regulation Questionnaire–Autonomous Motivation subscale (TSRQ-AM) in Japanese (score range 7-49, higher scores indicate greater autonomous motivation) [14].

Secondary outcomes included key physiological measures such as HbA_1c_, triglycerides, and body weight obtained during routine clinic visits. In addition, several psychological scales were administered for exploratory purposes: the Treatment Self-Regulation Questionnaire–Controlled Motivation subscale, Perceived Competence for Diabetes Scale [3], Japanese Summary of Diabetes Self-Care Activities [15], Self-Efficacy Scale for Diabetes Self-Care [16], Problem Areas in Diabetes scale [17], and World Health Organization–Five Well-Being Index [18].

Assessments were conducted at baseline, 6 weeks, 6 months, and 1 year after app implementation.

Although both self-reported continuation and objective use data were potentially available, these were intentionally excluded from the analysis. Our aim was to evaluate the impact of the implementation of such an app under real-world conditions rather than to establish a dose-response relationship. Therefore, we focused on outcomes irrespective of actual use intensity to avoid the tautological conclusion that those who used the app more frequently achieved greater improvements.

### Statistical Analysis

All statistical analyses were performed in R (version 4.5.1; R Foundation for Statistical Computing) using the *lme4* and *lmerTest* packages. Analyses were conducted with a four-step sequence:

First, we conducted a descriptive analysis of participant characteristics and each outcome at all time points.

Second, we created a visualization of time-course data for all psychological and physiological measures.

Third, we performed an exploratory trend analyses with linear mixed models (LMMs) to evaluate general trends in secondary psychological, behavioral, and clinical outcomes across the 4 assessment time points. The time variable was entered as an ordinal variable (0=baseline, 1=6 weeks, 2=6 months, and 3=1 year) to test for linear trends.

Fourth, we used longitudinal modeling of the primary outcome with a linear mixed-effects model to examine changes in TSRQ-AM scores over time, with random intercepts for participants to account for baseline differences. In the primary analysis, time was modeled as a continuous variable representing the actual months elapsed from baseline (coded as 0, 1.5, 6, and 12). Fixed effects included time (linear and quadratic terms), age, sex, employment status, family structure, baseline BMI, and baseline HbA_1c_. To capture potential nonlinear patterns, such as an inverted U-shaped trajectory (initial increase followed by a decline), both linear and quadratic time terms were included. Linear vs quadratic models were compared using likelihood ratio tests, and the final model retained both terms to capture potential nonlinear patterns such as peaks and declines. Marginal and conditional *R*^2^ values were calculated to quantify model fit. To verify the robustness of the findings against different time specifications, a sensitivity analysis was conducted in which time was treated as an ordinal numeric variable.

### Ethical Considerations

This study was conducted in accordance with the Declaration of Helsinki and approved by the ethical committee of Kobe City College of Nursing (approval 21,104-10). The study protocol was registered with the UMIN Clinical Trials Registry (UMIN000044874).

All participants provided written informed consent in person before enrollment and any study procedures. During the consent process, participants were informed of the study purpose, procedures, their right to withdraw at any time without penalty, and data confidentiality measures. Written consent for the publication of anonymized data was also obtained. Participants received a JP ¥3000 (US $25.50) reward for using the app for 7 days and JP ¥1000 (US $8.50) for each completed questionnaire.

To protect participant privacy, individuals were assigned unique study identification numbers, and their data were pseudonymized. The reidentification key was securely maintained and accessible only to the principal investigator. The anonymized research dataset used for analysis was stored in a password-protected environment and managed on password-secured systems to ensure data security.

## Results

### Participants

**Figure 1:**
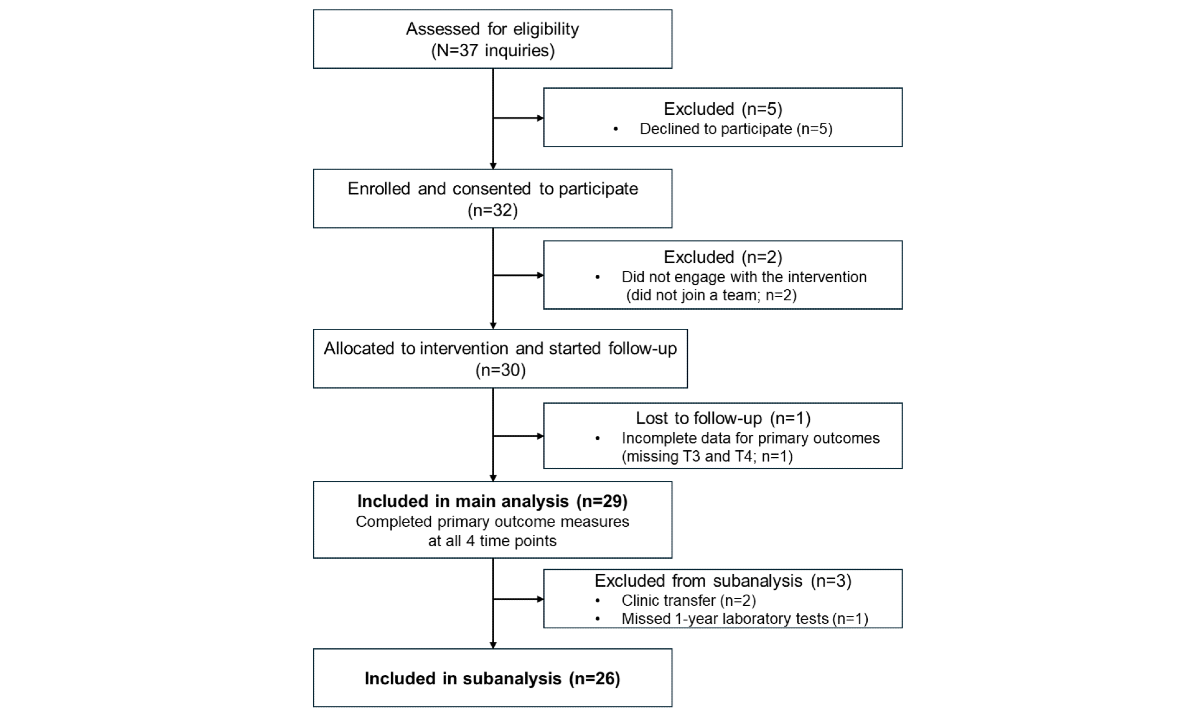
Flowchart of participant enrollment, allocation, follow-up, and analysis.

A total of 32 individuals with diabetes provided consent. Of these 32 participants, 2 (6.3%) did not join a team, and 1 (3.1%) withdrew from the study after the 6-week assessment, leaving 29 (90.6%) participants for the main analysis. Due to clinic transfers or missing laboratory assessments at the 1-year follow-up, HbA_1c_ and other laboratory data were available for 89.7% (26/29) of the participants and used in the subanalysis. The participant flow is shown in Figure 1.

Baseline characteristics are summarized in Table 1. The mean age was 61 (SD 10) years, and the mean HbA_1c_ at baseline was 6.96% (SD 0.51%).

**Table 1:** Baseline characteristics of study participants (N=29).

Characteristic					Values
**Continuous variables, mean (SD)**					
	Age (years)			61 (10)	
	BMI (kg/m^2^)			25.7 (5.2)	
	Hemoglobin A_1c_ (%)			6.96 (0.51)	
	Duration of diabetes (years)			11.8 (9.1)	
**Categorical variables, n (%)**					
	**Sex**				
		Male	19 (65.5)		
		Female	10 (34.5)		
	**Type of diabetes**				
		Type 2	28 (96.6)		
		Type 1	1 (3.4)		
	**Diabetic complications**				
		Present	7 (24.1)		
		Absent	22 (75.9)		
	**Employment status**				
		Full-time job	9 (31.0)		
		Part-time job	8 (27.6)		
		Homemaker	2 (6.9)		
		Unemployed	10 (34.5)		
	**Living arrangements**				
		Living alone	6 (20.7)		
		Living with spouse	17 (58.6)		
		Living with children or parents	4 (13.8)		
		Other	2 (6.9)		

### Changes in Psychological, Behavioral, and Clinical Outcomes

**Figure 2:**
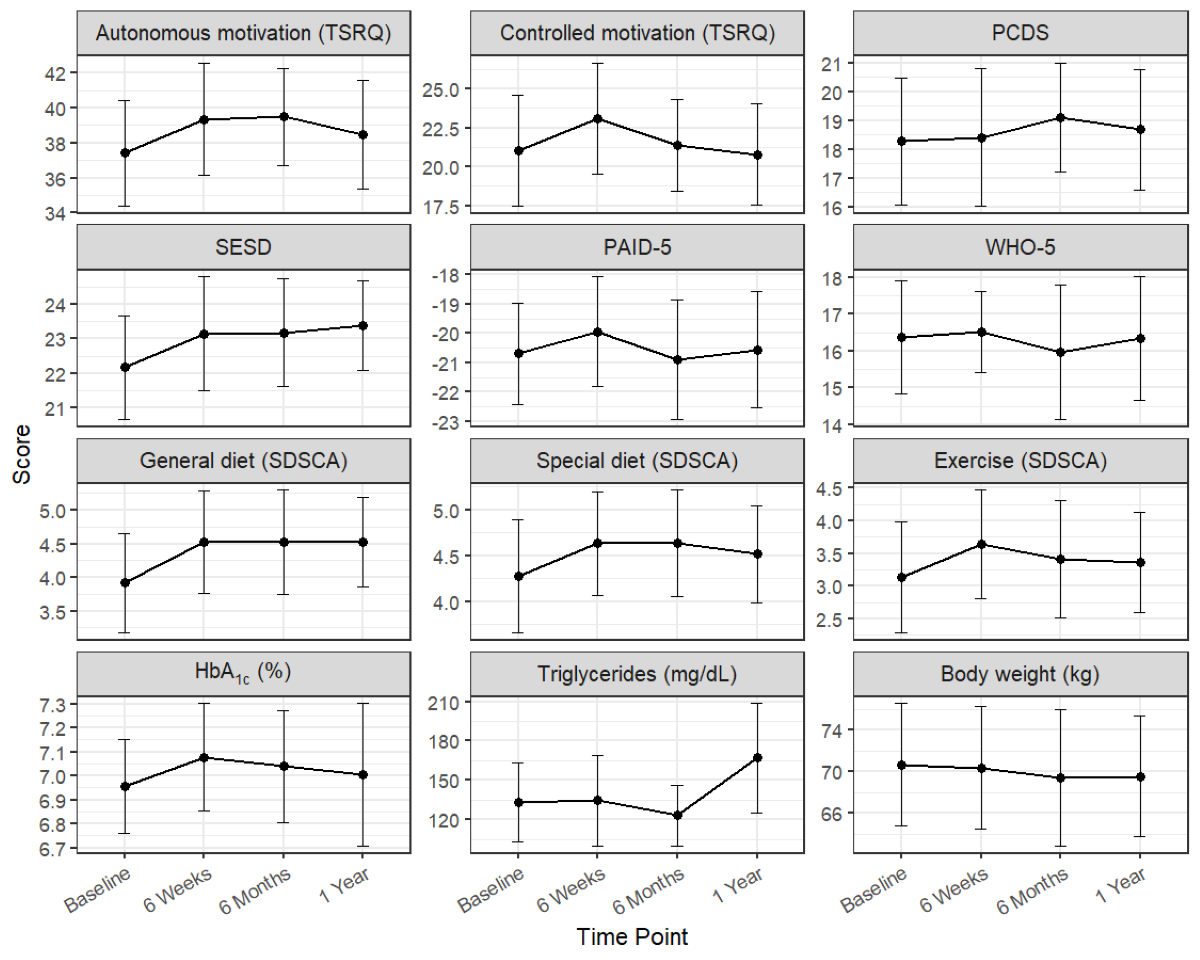
Mean changes in psychological, behavioral, and clinical outcomes over 4 assessment points. Dots represent estimated marginal means, and error bars indicate SEs. Refer to Table 2 for detailed descriptive statistics. HbA1c: glycated hemoglobin; PAID-5: Problem Areas in Diabetes scale; PCDS: Perceived Competence for Diabetes Scale; SDSCA: Summary of Diabetes Self-Care Activities; SESD: Self-Efficacy Scale for Diabetes Self-Care; TSRQ: Treatment Self-Regulation Questionnaire; WHO-5: World Health Organization–Five Well-Being Index.

Table 2 summarizes the descriptive statistics for all outcomes across the 4 assessment points. Figure 2 shows changes in group average values and 95% CIs for psychological and physiological measurements, whereas the spaghetti plot visualizing individual differences is shown in Multimedia Appendix 1.

**Table 2:** Changes in psychological, behavioral, and clinical measures across the 4 assessment points (baseline, 6 weeks, 6 months, and 1 year)^a^.

Measure and time point		Values, mean (SD)	Values, median (IQR)
**Autonomous motivation (TSRQ^b^; range 7-49)**			
	Baseline	37.4 (7.9)	38 (34-43)
	6 weeks	39.3 (8.4)	40 (36-46)
	6 months	39.5 (7.4)	40 (37-44)
	1 year	38.5 (8.2)	41 (35-44)
**Controlled motivation (TSRQ; range 7-49)**			
	Baseline	21.0 (9.3)	22 (13-28)
	6 weeks	23.1 (9.4)	24 (16-30)
	6 months	21.4 (7.7)	23 (16-27)
	1 year	20.8 (8.6)	21 (14-27)
**PCDS^c^ (range 4-28)**			
	Baseline	18.3 (5.8)	20 (15-22)
	6 weeks	18.4 (6.2)	18 (13-24)
	6 months	19.1 (4.9)	19 (16-24)
	1 year	18.7 (5.5)	19 (16-22)
**Self-efficacy (SESD^d^; range 8-32)**			
	Baseline	22.2 (3.9)	23 (18-25)
	6 weeks	23.1 (4.2)	23 (21-26)
	6 months	23.2 (4.1)	23 (20-26)
	1 year	23.4 (3.4)	23 (22-25)
**PAID-5^e^ (range 0-20)**			
	Baseline	9.3 (4.6)	9 (6-13)
	6 weeks	10.1 (4.9)	10 (6-14)
	6 months	9.1 (5.3)	10 (4-12)
	1 year	9.4 (5.2)	9 (6-13)
**WHO-5^f^ (range 0-25)**			
	Baseline	16.4 (4.0)	17 (14-19)
	6 weeks	16.5 (2.9)	17 (15-18)
	6 months	16.0 (4.8)	16 (14-19)
	1 year	16.3 (4.4)	16 (14-19)
**General diet (SDSCA^g^; range 0-7)**			
	Baseline	3.9 (1.9)	4 (3-5)
	6 weeks	4.5 (2.0)	5 (3-6)
	6 months	4.5 (2.0)	5 (3-6)
	1 year	4.5 (1.7)	4.5 (3-6)
**Special diet (SDSCA; range 0-7)**			
	Baseline	4.3 (1.6)	4.5 (3.5-5.5)
	6 weeks	4.6 (1.5)	5 (4-6)
	6 months	4.6 (1.5)	5 (4-5.5)
	1 year	4.5 (1.4)	4.5 (4-5.5)
**Exercise** (**SDSCA; range 0-7)**			
	Baseline	3.1 (2.2)	3 (1-5)
	6 weeks	3.6 (2.2)	3.5 (2-5.5)
	6 months	3.4 (2.4)	3.5 (1-5)
	1 year	3.4 (2.0)	3 (2-4.5)
**HbA_1c_^h^ (%)**			
	Baseline	7.0 (0.5)	6.9 (6.7-7.4)
	6 weeks	7.1 (0.6)	7.1 (6.7-7.3)
	6 months	7.0 (0.6)	6.9 (6.7-7.2)
	1 year	7.0 (0.7)	6.9 (6.5-7.2)
**Triglycerides (mg/dL)**			
	Baseline	133.3 (70.7)	125 (86.8-157.8)
	6 weeks	134.7 (71.8)	123 (92-163)
	6 months	123.2 (49.7)	118 (90-136.3)
	1 year	167.1 (86.3)	136 (108-195)
**Body weight (kg)**			
	Baseline	70.7 (15.5)	66.7 (60.8-80)
	6 weeks	70.4 (15.5)	66 (60.3-77)
	6 months	69.4 (16.3)	65.3 (60.2-76.8)
	1 year	69.6 (15.3)	68 (59-78)

^a^Clinical outcomes (hemoglobin A_1c_, triglycerides, and body weight) were available for 26 participants.

^b^TSRQ: Treatment Self-Regulation Questionnaire.

^c^PCDS: Perceived Competence for Diabetes Scale.

^d^SESD: Self-Efficacy Scale for Diabetes Self-Care.

^e^PAID-5: Problem Areas in Diabetes scale (short form).

^f^WHO-5: World Health Organization–Five Well-Being Index.

^g^SDSCA: Summary of Diabetes Self-Care Activities.

^h^HbA_1c_: hemoglobin A_1c_.

Exploratory trend analyses using LMMs revealed significant longitudinal improvements in specific outcomes (Table 3). Regarding psychological outcomes, self-efficacy for diabetes self-care increased significantly over the 1-year period (*b*=0.38; *P*=.02). Other psychological measures, including controlled motivation, did not show significant linear trends. Regarding behavioral outcomes, no significant linear trends were observed in self-care activities such as general and special diets or exercise habits. Regarding clinical outcomes, a significant reduction in body weight was observed (*b*=−0.39; *P*=.01), demonstrating a consistent weight loss trend throughout the study despite the lack of significant changes in HbA_1c_ (*P*=.40) or triglycerides (*P*=.14).

**Table 3:** Results of linear mixed models assessing time trends in psychological, behavioral, and clinical outcomes^a^.

Outcome			Unstandardized coefficient, B (SE; 95% CI)		*P* value
**Psychological outcomes**					
	Autonomous motivation (TSRQ^b^)	0.33 (0.3; –0.26 to 0.93)		.27	
	Controlled motivation (TSRQ)	−0.24 (0.39; –1.02 to 0.54)		.55	
	PCDS^c^	0.19 (0.26; –0.32 to 0.71)		.46	
	Self-efficacy (SESD^d^)	0.38 (0.16; 0.07 to 0.69)		*.02* ^e^	
	PAID-5^f^	−0.06 (0.19; –0.43 to 0.32)		.77	
	WHO-5^g^	−0.07 (0.2; –0.46 to 0.33)		.74	
**Self-care activities (SDSCA^h^)**					
	General diet	0.18 (0.11; –0.04 to 0.41)		.11	
	Special diet	0.07 (0.06; –0.04 to 0.19)		.22	
	Exercise	0.04 (0.13; –0.21 to 0.3)		.73	
**Clinical outcomes**					
	HbA_1c_^i^ (%)	0.03 (0.03; –0.04 to 0.1)		.40	
	Triglycerides (mg/dL)	6.91 (4.62; –2.34 to 16.16)		.14	
	Body weight (kg)	−0.39 (0.16; –0.70 to –0.08)		*.01* ^e^	

^a^Time was coded as an ordinal variable (0=baseline, 1=6 weeks, 2=6 months, and 3=1 years).

^b^TSRQ: Treatment Self-Regulation Questionnaire.

^c^PCDS: Perceived Competence for Diabetes Scale.

^d^SESD: Self-Efficacy Scale for Diabetes Self-Care.

^e^Statistical significance (*P*<.05).

^f^PAID-5: Problem Areas in Diabetes scale (5 items).

^g^WHO-5: World Health Organization–Five Well-Being Index.

^h^SDSCA: Summary of Diabetes Self-Care Activities.

^i^HbA_1c_: hemoglobin A_1c_.

### Longitudinal Trajectories of Autonomous Motivation (Primary Outcome)

A model comparison indicated that including a quadratic term for time provided a significantly better fit than a linear-only specification (*χ*^2^_1_=4.1; *P*=.04). Consequently, the quadratic model was adopted as the primary analysis.

**Figure 3:**
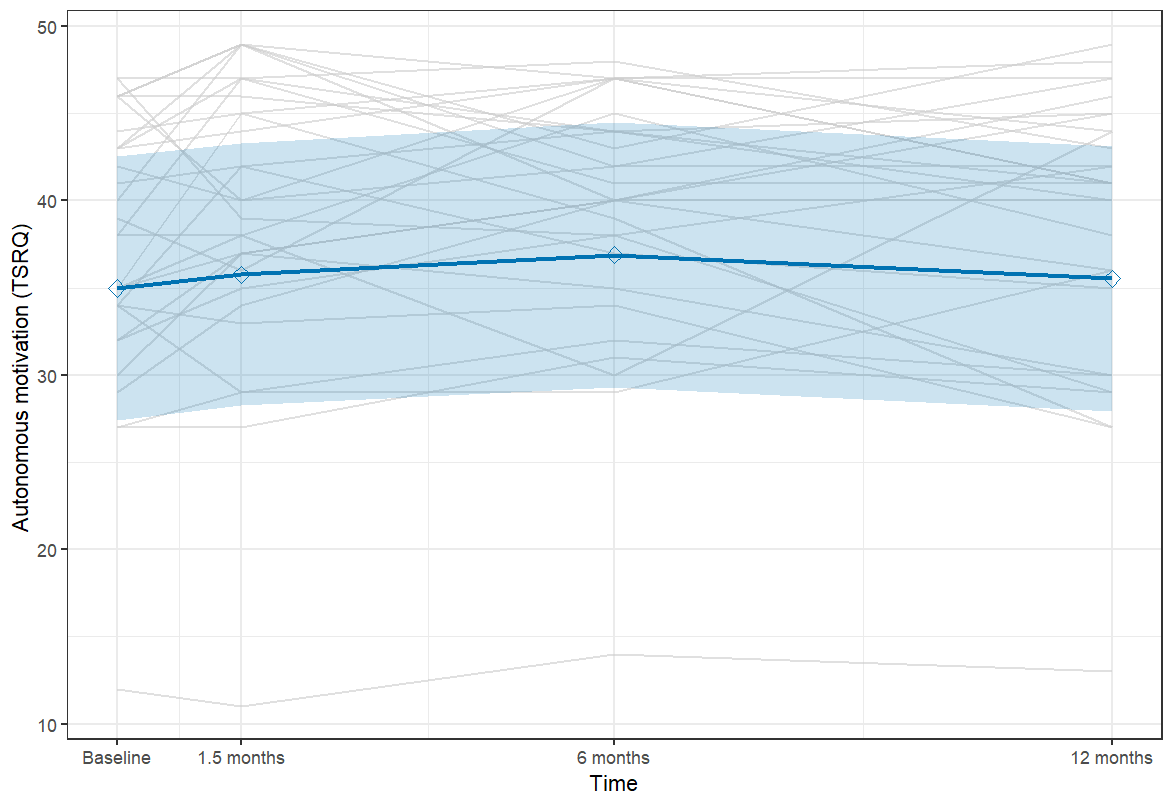
Model-estimated trajectories of Treatment Self-Regulation Questionnaire (TSRQ) scores on the autonomous motivation subscale across 4 assessment points (baseline, 6 weeks, 6 months, and 1 year). The solid line represents the estimated mean trajectory derived from a linear mixed-effects model, with the shaded area indicating the 95% CI. The gray lines depict individual participant trajectories.

As shown in Table 4, the linear effect of time was not statistically significant (*P*=.60), whereas there was a significant quadratic effect of time (*b*=−7.26; *P*=.045). This indicates an inverted curvilinear trajectory consistent with the pattern depicted in Figure 3, where autonomous motivation increased initially and then plateaued or declined. Descriptively, the mean score peaked at 6 months (39.5 vs 37.4 at baseline), representing an effect size of Cohen *d*=0.47. A sensitivity analysis treating time as ordinally coded time points (0-3) yielded consistent results, confirming a significant quadratic effect (Multimedia Appendix 2).

The fixed effects accounted for 46% of the variance in TSRQ-AM scores (marginal *R*^2^=0.46), whereas the full model including random intercepts explained 83% of the variance (conditional *R*^2^=0.83). Substantial between-participant variability was observed in baseline levels (mean random intercept variance 27.77, SD 5.27), with an intraclass correlation coefficient of 0.69, indicating that approximately 69% of the total variance was attributable to stable individual differences.

Among the covariates, higher baseline BMI was significantly associated with lower TSRQ-AM scores across time (*b*=−1.00; *P*=.001). No other demographic or clinical covariates reached statistical significance.

**Table 4:** Fixed-effects estimates from the linear mixed-effects model predicting autonomous motivation scores over 1 year^a^.

Variable	Coefficient (95% CI)	*P* value
Intercept	30.68 (–20.69 to 82.04)	.20
Time—linear	1.98 (–5.12 to 9.07)	.60
Time—quadratic	−7.26 (–14.36 to –0.16)	*.045* ^b^
Age	0.11 (–0.26 to 0.48)	.50
Sex: female	−3.88 (–10.33 to 2.58)	.20
Job: part-time	−1.36 (–8.93 to 6.21)	.70
Job: homemaker	−0.99 (–16.3 to 14.32)	.90
Job: unemployed	1.81 (–4.74 to 8.35)	.60
Baseline BMI	−1.00 (–1.55 to –0.46)	*.001* ^b^
Baseline HbA_1c_^c^	3.46 (–2.35 to 9.26)	.20
Family: living with spouse	5.71 (–1.74 to 13.16)	.12
Family: living with children or parents	3.56 (–5.5 to 12.62)	.40
Family: other	2.97 (–9.16 to 15.1)	.60

^a^Time was coded as a continuous variable representing actual months (0, 1.5, 6, and 12) and modeled using orthogonal polynomials to avoid multicollinearity. Consequently, the coefficients for linear and quadratic terms represent orthogonal contrasts rather than raw monthly changes. Employment status and family structure were treated as categorical variables, with “full-time employment” and “living alone” as reference categories, respectively. The estimates shown are fixed effects; random intercepts (not shown) account for individual baseline differences in Treatment Self-Regulation Questionnaire–Autonomous Motivation score.

^b^Statistical significance (*P*<.05).

^c^HbA_1c_: hemoglobin A_1c_.

## Discussion

### Principal Findings

The primary finding of this study was the curvilinear trajectory of autonomous motivation, which peaked at 6 months and then declined, although it remained above baseline after 1 year of implementing use of a commercially available, team-based gamification app. There was no significant change in HbA_1c_, suggesting that psychological benefits may occur independently of physiological improvements.

### Motivational Trajectories and Individual Variability

The observed trajectories of motivation can be interpreted within the framework of SDT [4]. Autonomous motivation, in particular, followed a curvilinear trajectory, increasing substantially and persisting above baseline even after 1 year. This pattern underscores the unique role of autonomous motivation in sustaining health behaviors and is consistent with prior SDT-based studies linking autonomous forms of regulation with long-term adherence to self-care [2,3,19]. However, our findings extend this literature by identifying a significant nonlinear, curvilinear pattern (*P*=.045) that peaked at 6 months before modestly declining, providing a novel and critical insight into the temporal dynamics of app-supported motivation. In contrast, descriptive data suggested that controlled motivation increased at 6 weeks but returned to baseline levels thereafter. This transient elevation likely reflects a temporary sense of pressure to meet team expectations—an external regulatory process [20] that is, by nature, short-lived. However, no significant deterioration in psychological well-being (World Health Organization–Five Well-Being Index) or diabetes-related distress (Problem Areas in Diabetes scale) was observed during this period. This suggests that the initial social pressure did not result in psychological harm. Few mHealth or gamification studies have tracked motivational processes over a full year, making these findings valuable in extending evidence beyond the short-term effects typically reported.

At the same time, the mixed-effects model revealed substantial variability in baseline autonomous motivation (mean random intercept variance 27.8, SD 5.3). Further inspection of individual trajectories highlighted considerable heterogeneity: some participants showed early declines, whereas others maintained or even enhanced their motivation. A plausible contributor is differential engagement [21] with the app, such as frequency of log-ins or postings. We intentionally did not systematically assess this engagement factor in this study to avoid the tautological conclusion that patients who used the app more frequently achieved better outcomes. Instead, our aim was to examine the outcomes when the app was merely implemented, regardless of how intensively participants used it. Participants were only asked to download and try the app for at least 7 days, with the option to discontinue thereafter. Even under these conditions, the overall trend still indicated psychological benefits for many participants, suggesting that peer presence and social comparison [13] may exert influence even without intensive engagement. It is also possible that the act of prescribing the app or the decision to accept it itself fostered a sense of autonomy. This raises the possibility that digital tools may offer psychological benefits through the very act of adoption, not solely through intensive use.

### Comparison With In-Person Peer Support

Our findings are consistent with those of previous research on the distinct effects of peer support on behavioral and physiological outcomes. While some real-world peer support initiatives have demonstrated modest HbA_1c_ reductions [7,22], others, particularly those involving participants with well-controlled baseline glycemic levels, have not observed significant metabolic improvements despite behavioral gains [23]. For instance, Yin et al [24] demonstrated that patients with well-controlled blood glucose can improve self-management behaviors through peer support without necessarily altering glycemic control. Our study corroborates this pattern, suggesting that while enhancing autonomous motivation is critical for adherence, its independent impact on HbA_1c_ may be modest relative to pharmacological therapy [25], especially in a population with a mean baseline HbA_1c_ of 6.96% (SD 0.51%).

However, unlike traditional in-person peer support, which faces logistical barriers [6,9], the digital nature of the intervention in this study allowed for the preservation of emotional connection and practical assistance independent of time and place. This flexibility in changing teams was a feature valued by participants in our prior qualitative work [13].

With regard to secondary outcomes, while enhancements in self-reported diet and exercise were not statistically significant, our longitudinal analysis confirmed a substantial reduction in body weight. This discrepancy suggests that participants may have incorporated subtle yet effective lifestyle modifications that were not consciously perceived as major behavior changes. This finding is consistent with previous research indicating that mHealth and peer support interventions are particularly effective in promoting simple, sustainable lifestyle modifications [11,26].

### Clinical Implications

The findings of this study offer several practical insights for integrating digital health tools into diabetes care.

First, in terms of the magnitude of the effect, the observed increase in autonomous motivation (1-2 points on the TSRQ-AM) should be considered within the context of behavioral science and cost-effectiveness. Although meta-analyses suggest that the effect sizes of health behavior interventions are usually modest, approximately *d*=0.64 [27], this study achieved a Cohen *d* of approximately 0.47 at the 6-month follow-up. While this represents a small to medium effect size, it is consistent with the range reported in other studies [27-29]. The clinical significance of this finding is further emphasized by the fact that the intervention was delivered at no cost and without the direct involvement of health care providers. Thus, even the modest motivational gains make this a valuable, scalable solution for long-term health behavior management when viewed from a population health perspective.

Second, our findings identify the 6-month mark as a critical opportunity for re-engagement. Engagement peaked at this point and then gradually declined, suggesting that clinicians should schedule a brief “motivational check-in.” If engagement is waning, this moment is well suited for proposing “team reorganization” as joining a new team may refresh social dynamics and restore a sense of novelty.

Finally, interindividual variability in this study indicated that lower motivation among patients with higher baseline BMI identifies a group needing additional support. This inverse association is consistent with evidence linking higher BMI to attrition and poor adherence, potentially due to reduced reward sensitivity [30] and greater reliance on less sustainable, externally driven motivation [31]. For these patients, clinicians should consider a hybrid strategy that combines the app with regular face-to-face counseling rather than relying on the digital tool alone [32,33].

### Limitations and Future Research Directions

Several limitations of this study should be acknowledged.

First, the single-arm, pretest-posttest design without a control group limits causal inference. The observed changes may be attributable not only to the intervention but also to natural maturation, testing effects, or regression to the mean. Second, the small sample size (N=29), which predominantly comprised older adults with type 2 diabetes, reduces statistical power and limits the generalizability of the findings. However, we note that the use of LMMs with 4 repeated measures allowed for the estimation of individual trajectories, providing preliminary evidence on the significant quadratic trend in our primary psychological outcome (TSRQ-AM score). Participants were also volunteers, potentially introducing selection bias toward individuals with greater baseline motivation or digital literacy. Third, psychological outcomes were assessed solely through self-report questionnaires, which may be susceptible to social desirability or recall bias. Finally, the intervention included multiple components (team structure, peer support, social comparison, and gamification rewards), making it difficult to disentangle which elements were most responsible for the observed changes. Further research with larger, randomized samples and process evaluations is needed to elucidate causal mechanisms and optimize intervention design.

### Conclusions

In conclusion, this formative longitudinal study provides preliminary evidence on the 1-year nonlinear trajectory of autonomous motivation following the introduction of a team-based gamification app. The observed curvilinear pattern suggests that motivation may peak at around 6 months, underscoring the importance of adaptive intervention designs to maintain engagement over time. Furthermore, this study demonstrated a significant reduction in body weight, suggesting potential physiological benefits.

## Appendix

Multimedia Appendix 1 Changes in psychological, behavioral, and clinical outcomes over 1 year.

Multimedia Appendix 2 Sensitivity analysis using ordinally coded time points (0-3): multivariable linear mixed-effects model of autonomous motivation (Treatment Self-Regulation Questionnaire–Autonomous Motivation subscale) over 1 year.

## Abbreviations

HbA_1c_: hemoglobin A_1c_
LMM: linear mixed model
mHealth: mobile health
SDT: self-determination theory
TSRQ-AM: Treatment Self-Regulation Questionnaire–Autonomous Motivation subscale

## Data Availability

The datasets generated or analyzed during this study are not publicly available due to participant privacy and ethical restrictions but are available from the corresponding author on reasonable request.
