# Supplementary material for: Autonomous Motivation Trajectory Following Adoption of a Team-Based Gamification App Among Adults With Diabetes: 1-Year Formative Longitudinal Study
Source: JMIR Form Res. 2026 Feb 19;10:e87236. doi: 10.2196/87236 (PMC12963977; doi:10.2196/87236)
Supplement: Multimedia Appendix 1 [file formative_v10i1e87236_app1.pdf]

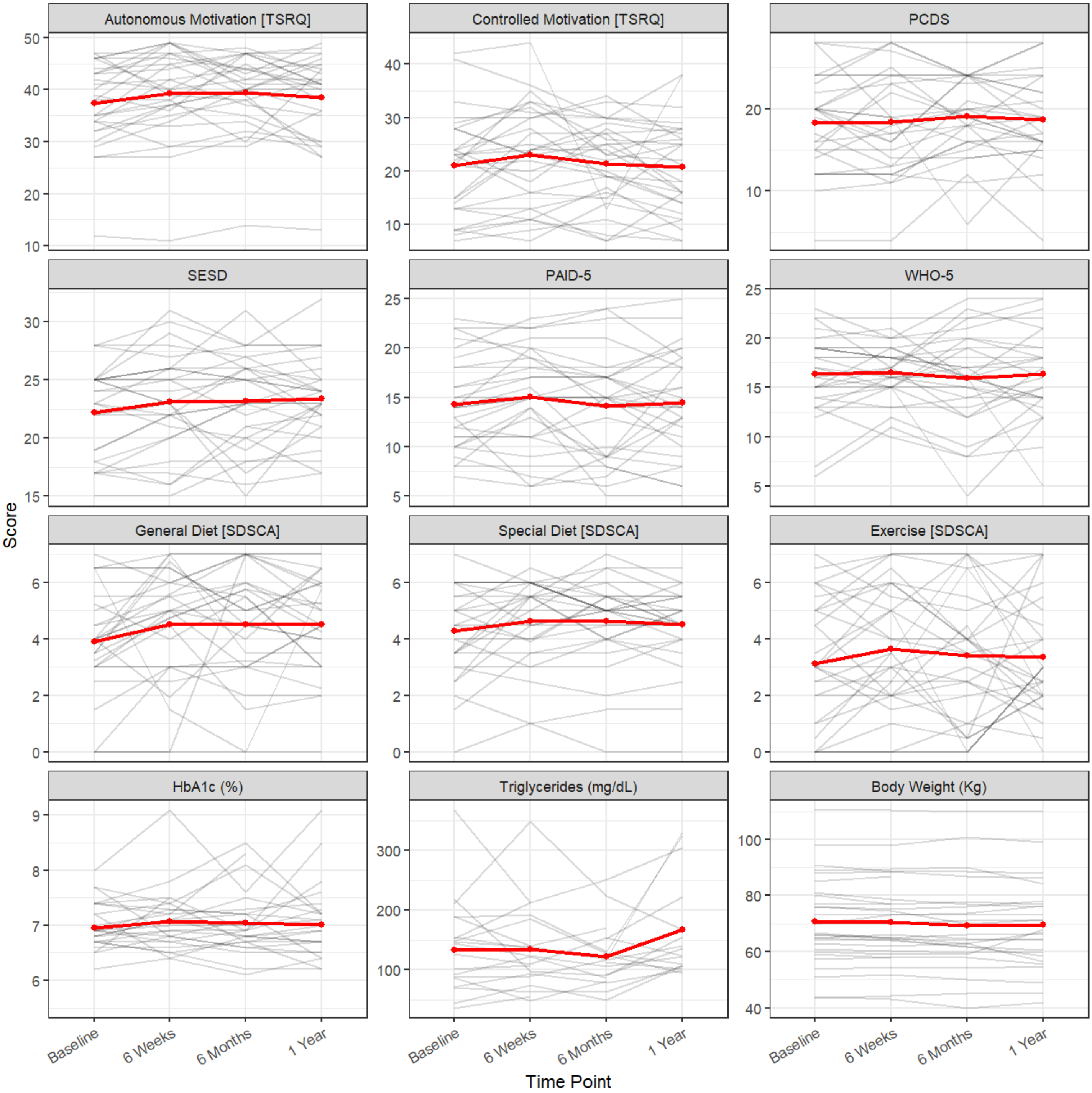

Figure S1. Changes in Psychological, Behavioral, and Clinical Outcomes Over One Year.

Grey lines represent individual participant trajectories (N = 29), while red points and lines indicate the estimated mean at each assessment point (Baseline, 6 Weeks, 6 Months, and 1 Year).

Outcomes include psychological scales (Treatment Self-Regulation Questionnaire for Diabetes, TSRQ; Self-Efficacy Scale for Diabetes Self-care, SESD; Perceived Competence for Diabetes Scale, PCDS; Problem Areas in Diabetes Scale, PAID-5; WHO-5 Well-Being Index), behavioral measures (Summary of Diabetes Self-Care Activities, SDSCA: General Diet, Special Diet, and Exercise subscales), and clinical indicators (HbA1c, triglycerides, body weight).
