# Supplementary material for: Autonomous Motivation Trajectory Following Adoption of a Team-Based Gamification App Among Adults With Diabetes: 1-Year Formative Longitudinal Study
Source: JMIR Form Res. 2026 Feb 19;10:e87236. doi: 10.2196/87236 (PMC12963977; doi:10.2196/87236)
Supplement: Multimedia Appendix 2 [file formative_v10i1e87236_app2.pdf]

Supplementary Table 1. Sensitivity analysis using ordinally coded time points (0–3): multivariable linear mixed-effects model of Autonomous Motivation (TSRQ-AM) over one year

Variable	Coefficient	95% CI	p-value	Significance
(Intercept)	30.68	(-20.69, 82.04)	0.23	
Time (linear)	4.03	(-3.00, 11.05)	0.26	
Time (quadratic)	-7.89	(-14.92, -0.87)	**0.028**	*
Age	0.11	(-0.26, 0.48)	0.53	
Sex: Female	-3.88	(-10.33, 2.58)	0.22	
Job: Part-time	-1.36	(-8.93, 6.21)	0.71	
Job: Homemaker	-0.99	(-16.30, 14.32)	0.89	
Job: Unemployed	1.81	(-4.74, 8.35)	0.57	
BMI	-1.00	(-1.55, -0.46)	**0.001**	**
Baseline HbA1c	3.46	(-2.35, 9.26)	0.23	
Family: Married Couple	5.71	(-1.74, 13.16)	0.13	
Family: Two Generations	3.56	(-5.50, 12.62)	0.42	
Family: Other	2.97	(-9.16, 15.10)	0.61	

Abbreviations: TSRQ-AM = Treatment Self-Regulation Questionnaire for Diabetes–Autonomous Motivation; CI = Confidence Interval; BMI = Body Mass Index; HbA1c = Glycated Hemoglobin.

Note: $^{*}p < 0.05$, $^{**}p < 0.01$.
This analysis was conducted as a sensitivity check for the main model presented in Table 4. Time was coded as an ordinal numeric variable (0 = baseline, 1 = 6 weeks, 2 = 6 months, 3 = 1 year). The significant quadratic effect observed here (p=.028) confirms that the inverted U-shaped trajectory of autonomous motivation is robust regardless of time coding.
